# Ketamine analgesia for inflammatory pain in neonatal rats: a factorial randomized trial examining long-term effects

**DOI:** 10.1186/1744-9081-4-35

**Published:** 2008-08-07

**Authors:** Cynthia R Rovnaghi, Sarita Garg, Richard W Hall, Adnan T Bhutta, K JS Anand

**Affiliations:** 1 Pain Neurobiology Laboratory, Arkansas Children's Hospital Research Institute, Little Rock, Arkansas 72202, USA; 2 Department of Surgery, College of Medicine, University of Arkansas for Medical Sciences, Little Rock, Arkansas 72205, USA; 3 Department of Pediatrics and Center for Translational Neuroscience, College of Medicine, University of Arkansas for Medical Sciences, Little Rock, Arkansas 72205, USA; 4 Department of Pediatrics, College of Medicine, University of Arkansas for Medical Sciences, Little Rock, Arkansas 72205, USA; 5 Departments of Pediatrics, Anesthesiology, Pharmacology, Neurobiology & Developmental Sciences, College of Medicine, University of Arkansas for Medical Sciences, Little Rock, Arkansas 72205, USA

## Abstract

**Background:**

Neonatal rats exposed to repetitive inflammatory pain have altered behaviors in young adulthood, partly ameliorated by Ketamine analgesia. We examined the relationships between protein expression, neuronal survival and plasticity in the neonatal rat brain, and correlated these changes with adult cognitive behavior.

**Methods:**

Using Western immunoblot techniques, homogenates of cortical tissue were analyzed from neonatal rats 18–20 hours following repeated exposure to 4% formalin injections (F, N = 9), Ketamine (K, 2.5 mg/kg × 2, N = 9), Ketamine prior to formalin (KF, N = 9), or undisturbed controls (C, N = 9). Brain tissues from another cohort of rat pups (F = 11, K = 12, KF = 10, C = 15) were used for cellular staining with Fos immunohistochemistry or FluoroJade-B (FJB), followed by cell counting in eleven cortical and three hippocampal areas. Long-term cognitive testing using a delayed non-match to sample (DNMS) paradigm in the 8-arm radial maze was performed in adult rats receiving the same treatments (F = 20, K = 24, KF = 21, C = 27) in the neonatal period.

**Results:**

Greater cell death occurred in F vs. C, K, KF in parietal and retrosplenial areas, vs. K, KF in piriform, temporal, and occipital areas, vs. C, K in frontal and hindlimb areas. In retrosplenial cortex, less Fos expression occurred in F vs. C, KF. Cell death correlated inversely with Fos expression in piriform, retrosplenial, and occipital areas, but only in F. Cortical expression of glial fibrillary acidic protein (GFAP) was elevated in F, K and KF vs. C. No significant differences occurred in Caspase-3, Bax, and Bcl-2 expression between groups, but cellular changes in cortical areas were significantly correlated with protein expression patterns. Cluster analysis of the frequencies and durations of behaviors grouped them as exploratory, learning, preparatory, consumptive, and foraging behaviors. Neonatal inflammatory pain exposure reduced exploratory behaviors in adult males, learning and preparatory behaviors in females, whereas Ketamine ameliorated these long-term effects.

**Conclusion:**

Neuroprotective effects of Ketamine attenuate the impaired cognitive behaviors resulting from pain-induced cell death in the cortical and hippocampal fields of neonatal rats. This cell death was not dependent on the apoptosis associated proteins, but was correlated with glial activation.

## Background

Exposure to adverse experiences in early life alters brain function and behavior in childhood because of plasticity in the immature nervous system [1]. Experiences during early development will alter neuronal activity patterns and the functional wiring of immature neurons. Epidemiological studies suggest that adverse experiences in the perinatal or neonatal periods may be associated with atypical behavior or emotional problems during childhood [2], such as anxiety, depression [2,3], or even suicidal tendencies [4,5].

Prolonged or repetitive pain occur during critical periods of brain development in preterm neonates [6]. Rapid brain growth, synaptogenesis, expression of excitatory receptors [7] and developmentally regulated neuronal cell death [8] also occur at this time, which may explain why repetitive neonatal pain persistently alters pain processing, in rats [9], mice [10], and humans [11-13]. Prolonged treatment of infant rats with high doses of analgesic or anesthetic agents also triggers widespread neurodegeneration in their brain [14]. It is important, therefore, to study the mechanisms by which repetitive pain or prolonged anesthetic exposure alter development in the neonatal brain, through factors altering cell survival, neuronal activity, or plasticity. We are the first to report 26 rat behaviors assessing cognitive deficits in a delayed non-match to sample (DNMS) paradigm, correlated with the expression of various cellular proteins that regulate the mechanisms of cell death and plasticity.

## Methods

All experiments were consistent with National Institutes of Health animal use guidelines and approved by the local Animal Care and Use Committee.

### Animals

Timed pregnant Long-Evans hooded rats were moved to cages with increased bedding (3" layer) in a noise-free parturition room on embryonic (E) day 18 (E18). Pregnant females were handled daily by the same personnel who provided the perinatal animal husbandry. Birthing cages were not changed after nesting occurred and the litters were left undisturbed. Environmental noise, changes in temperature or humidity, or changes in husbandry staff were strictly controlled, while maintaining a 12:12-h light-dark cycle, with food and water *ad lib*. On the day of birth (P0), rat pups were randomly cross-fostered and culled to eight pups per dam.

Rat pups were randomly assigned to undisturbed controls (C), or receiving subcutaneous injections of 4% formalin (F), Ketamine and formalin (KF), or Ketamine alone (K). Formalin (5 μl) was injected at hourly intervals into each paw once daily from P1 to P4; Ketamine (2.5 mg/kg × 2) was injected under the interscapular skin, 5 minutes before the first and third formalin injections for acquiring cellular data. All rats for the neonatal studies were sacrificed on P5 to harvest brain tissues at the peak period of neuronal cell death, 18–20 hrs after the last injection [15]. Brain tissues from an initial cohort of rat pups (C = 15, F = 11, KF = 10, K = 12) were used for Fos immunohistochemistry and FluoroJade-B (FJB) staining, whereas brain tissues from 9 additional pups in each group (C, F, KF, K) were used for Western immunoblot analyses. The final cohort (C = 27, F = 20, KF = 21, K = 24) was raised for long-term cognitive testing using a delayed non-match to sample paradigm in the radial arm maze.

### Cellular staining

Infant rats from all groups were anaesthetized with ether and perfused with fresh, ice-cold 4% paraformaldehyde. Brains were harvested, immersed in paraformaldehyde, then 20% sucrose, and frozen in cryoprotectant. Cryostat sections (20 μm) were mounted on positively charged slides and stained with Fos antibody (Oncogene, Inc.) or FluoroJade-B (FJB) (Histo-Chem). FJB staining, a marker for neurodegeneration, and Fos immunohistochemistry were conducted as reported previously [9,16]. Two observers, blinded to study group assignment, counted the stain-positive cells in the cortex, hippocampus, amygdala, thalamus, hypothalamus, and habenula. All cell counts were repeated for differences > 8% and statistical analyses included analysis of variance (ANOVA), followed by Dunn's or Tukey-Kramer *post hoc *tests, with significance set at p < 0.01.

### Western immunoblot analysis

Fresh tissue for protein extraction was dissected in cortical and subcortical regions, frozen in liquid nitrogen, and stored at -80°C. Protein was extracted by adding 4 volumes of Tri Reagent (Molecular Research Center, Inc.), homogenized using PowerGen 125 (Fisher Scientific), and analyzed using the Micro-BCA protein assay kit, with optical densities read at 562l. The Mini-Trans Blot electrophoresis and transfer set-up was used, with 7–9 samples in each gel and pre-stained SDS-Page standards (Bio-Rad, Inc.). Pre-cast 10% separation and 4% stacking SDS-Page gels were used for electrophoresis at 150V in standard buffer. For separation times exceeding one hour, electrophoresis units were kept refrigerated at 4°C.

The Silver Stain Plus kit (Bio-Rad, Inc.) was used to confirm protein integrity and the evenness of protein concentration loading. Immune-Blot PVDF membrane (0.2 μm pore size) was used for transfers, after blocking non-specific antibody binding. Specific dilution and incubation times were determined for each lot of the primary antibodies, which included: GFAP (glial fibrillary acidic protein, 1:5000 dilution, DAKO, Inc.), Caspase-3 (1:2000 dilution, Calbiochem), PARP (poly ADP-ribose polymerase, 1:2000 dilution, Cell Signaling, Inc.), Bax and Bcl-2 (1:2000 dilution for each, R&D Systems, Inc.). Immun-Star Chemiluminescent Protein Detection Systems were used as the secondary antibody (1:3000 dilution, Bio-Rad, Inc.). Membranes were exposed to Fuji film for 5–10 minutes. Specific protein levels were measured by densitometry, with integrated density values adjusted for area obtained from ChemiImager 5500 imaging system with AlphaEaseFC software (Alpha Innotech Corp.). Data analysis used ANOVA, with significance levels at p < 0.05.

### Radial arm maze test

Spatial learning and memory were measured using standard procedures for the radial 8-arm maze (RAM) in adult rats [17]. Prior to testing, rats (N = 27, 24, 21, 20 from C, K, KF, F groups, respectively) were diet-restricted to 85% of baseline body weight and received training for 5 days [18]. A delayed non-match to sample (DNMS) paradigm was used with initial exposure to the radial maze (4 lanes open) followed by a second exposure (all 8 lanes open) after intervals of 1 hour (on P63) or 3 hours (on P64). Within the time required to consume all bait, the frequency and duration of behaviors, including rearing, re-entry into bait eaten arm, time required to eat bait, and incomplete consumption of bait were measured. Data analysis used factorial ANOVA, with significance levels at p < 0.05.

### Data analyses

In addition to the ANOVAs cited above, Pearson's correlations were used to compare site specific cortical cellular activation and death to the levels of apoptosis gene products present in the cortex. Individual data points were analyzed only once using the statistical methods noted above, thus corrections for repeated statistical testing were not required. All data are presented as Mean ± SEM (standard error of the mean). The behavioral data were analyzed using Statistical Analysis Software (SAS). Initial data analyses were performed using ANOVA and factorial ANOVA, with significance levels set at p < 0.05. Oblique centroid component cluster analysis was performed to show differences between the treatment groups and genders based on cognitive behaviors. These behaviors were separated into 5 clusters; exploratory, learning, preparatory, consumptive and foraging behaviors, calculated based on the unweighted averages of the standardized variables. The bar graphs were plotted as variation of means by time between the treatment groups.

## Results

### Cortical expression of neuronal activation or cell death

**Figure 1:**
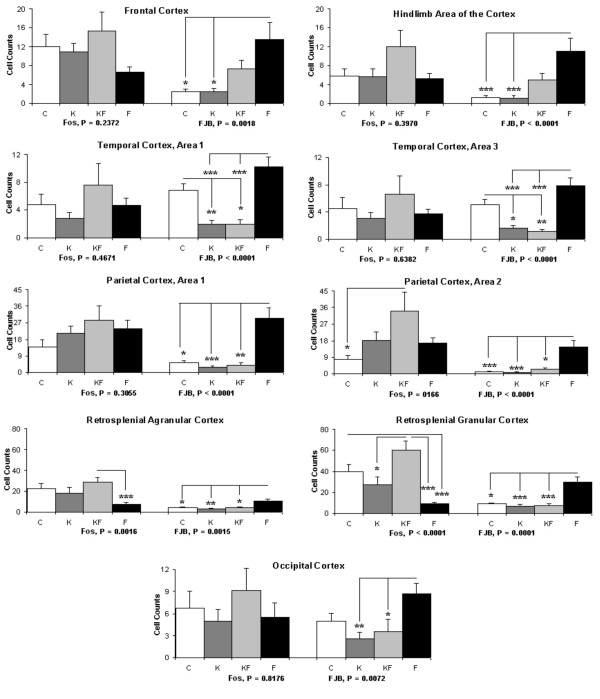
**Number of cells in cortical areas showing Fos expression and cell death (FJB staining)**. Control (C), Ketamine (K), Ketamine and Formalin (KF), Formalin (F). Bars indicate Mean ± SEM; *** P < 0.001; ** P < 0.01; * P < 0.05.

**Figure 2:**
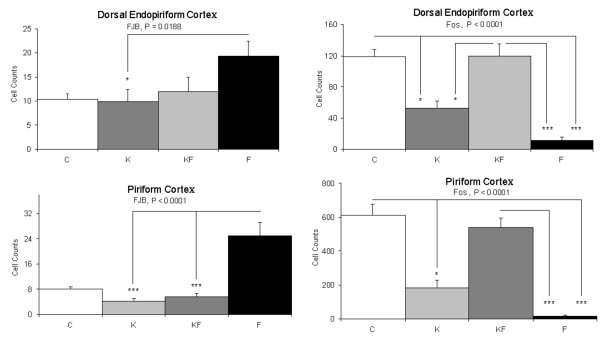
**Number of cells in the pirifom and dorsal endopiriform showing Fos expression and cell death**. Control (C), Ketamine (K), Ketamine and Formalin (KF), Formalin (F). Bars indicate Mean ± SEM; *** P < 0.001; * P < 0.05.

The cellular markers for activation (Fos) and cell death (FJB) were compared between C, K, KF, and F groups. Greater cell death occurred in many cortical areas of the F animals, compared to C, K in the frontal cortex and hindlimb area, compared to K, KF in the temporal (areas 1 and 3) and occipital cortex, and compared to C, K, and KF in the parietal (areas 1 and 2), granular and agranular retrosplenial cortex (fig. 1). Retrosplenial areas of the cortex showed significantly reduced Fos expression in F, compared with KF in the agranular area (p < 0.001), with C and KF in the granular area (p < 0.001). Differences in the limbic system, which included the dorsal endopiriform cortex showed no differences in cell death, but significantly lower Fos expression in F (vs. C, K, KF). In the piriform cortex, the F group showed greater cell death (vs. K, KF) and lower Fos expression (vs. C, K, KF) (fig. 2). Inverse correlations between Fos expression and cell death occurred only the F group, particularly significant in the piriform cortex (r = -0.7, p = 0.02), granular retrosplenial cortex (r = -0.7, p = 0.03), and occipital cortex (r = -0.6, p = 0.05).

### Protein expression in cortical areas

**Figure 3:**
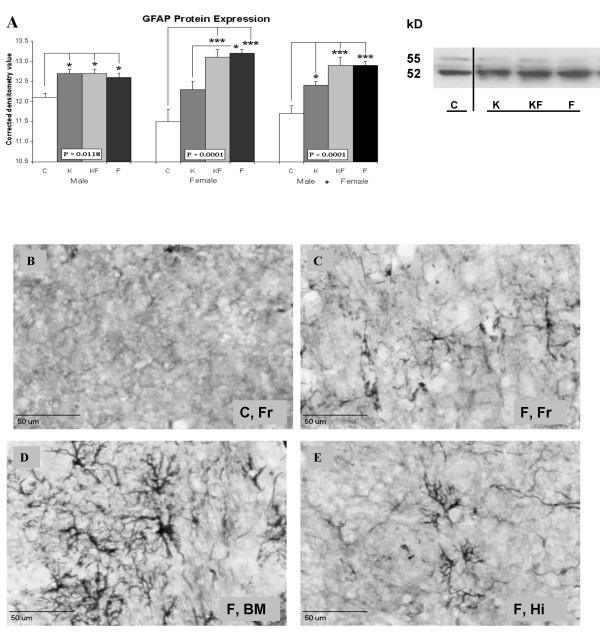
**Western immunoblot assay (A) and immunohistochemistry (B-E) showing GFAP (glial fibrillary acidic protein)**. Control (C), Ketamine (K), Ketamine and Formalin (KF), Formalin (F). Photomicrographs show GFAP staining in the C group: Frontal cortex (B) and the F group: Frontal cortex (C), basomedial amygdaloid nucleus (D), hippocampus (E). Bars indicate Mean ± SEM; *** P < 0.001; * P < 0.05; kD = kilodaltons; line bar = 50 μm.

**Figure 4:**
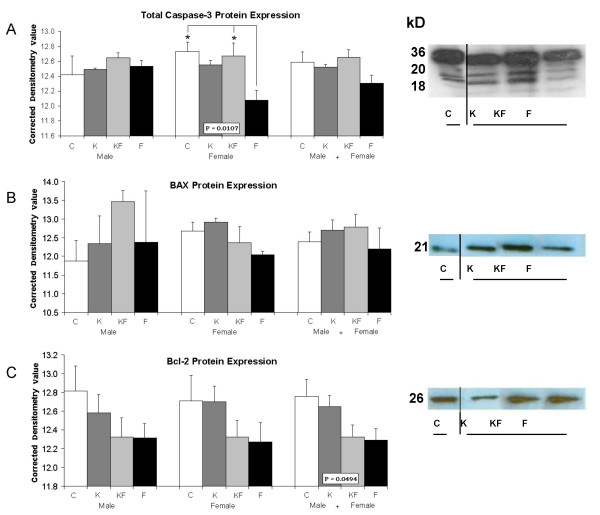
**Western immunoblot assays showing Caspase-3 (A), Bax (B), and Bcl-2 (C) protein expression**. Control (C), Ketamine (K), Ketamine and Formalin (KF), Formalin (F). Bars indicate Mean ± SEM; * P < 0.05; kD = kilodaltons.

Cell death mechanisms were further investigated using antibodies for apoptotic proteins or glial activation. Significantly greater glial activation indicated by GFAP expression occurred in formalin-treated groups (F, KF), with smaller increases in K (fig. 3A). Examples of glial activation in the frontal cortex of C (fig. 3B) and F (fig. 3C) are compared, in contrast with glial morphology in subcortical areas such as the basomedial amygdala (fig 3D) and hippocampus in the F group (fig. 3E). Relatively greater GFAP expression occurred in the formalin-injected females than male rats (vs. C, p < 0.001, fig. 3A). Cortical expression of caspase-3 was diminished in females from F (vs. C/KF, p < 0.05, fig. 4A), but no differences occurred for Bax or Bcl-2 expression (fig. 4B, 4C) between the randomized groups.

### Cellular changes, protein expression and cortical areas

**Figure 5:**
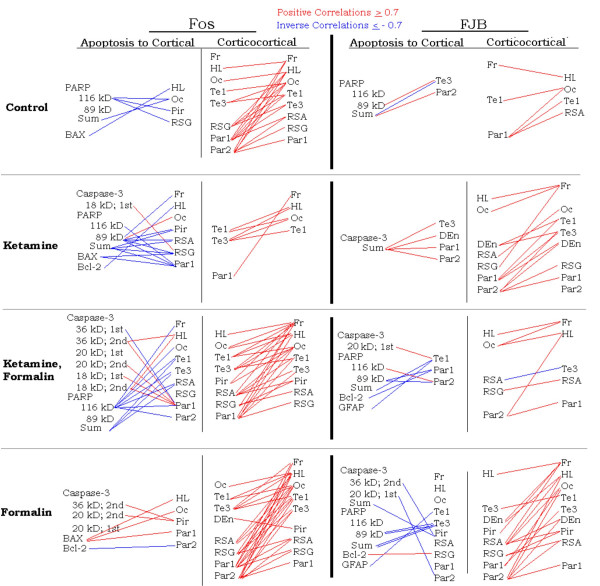
**Correlations between apoptotic protein expression and cell counts of neurons stained with Fos or FJB**. Positive correlations (red lines) Pearson r ≥ 0.7 and inverse correlations (blue lines) Pearson r ≤ -0.7 are depicted. Fr = frontal, HL = hindlimb, Oc = occipital, Te1 = temporal area 1, Te3 = temporal area 3, RSA = retrosplenial agranular, RSG = retrosplenial granular, Par1 = parietal area 1, Par2 = parietal area 2, Pir = pirifom cortex, DEn = dorsal endopiriform, kD = kilodaltons.

Protein expression associated with inflammatory pain and/or Ketamine analgesia may differentially alter processes such as cellular activation or cell death in the different cortical areas. Cellular changes in specific cortical areas were correlated significantly with each other and with protein expression patterns, but only the strong correlations (r ≥ 0.7, or r ≤ -0.7) were considered to be developmentally significant. Robust correlations for Fos expression between various cortical areas occurred within C, KF, and F, but occurred sparsely in K (fig. 5). Most of the proteins mediating cell death were positively correlated with cortical Fos expression in F, but inversely correlated in C, K, and KF (fig. 5). These proteins were inversely correlated with cell death in cortical areas from KF and F, with only sparse correlations in C and K. The extent of cell death occurring cortical areas was frequently correlated with other cortical areas in F, but fewer correlations occurred in C, K, and KF (fig. 5) because of the diminished cell death within these groups.

### Behavioral cluster analyses

**Figure 6:**
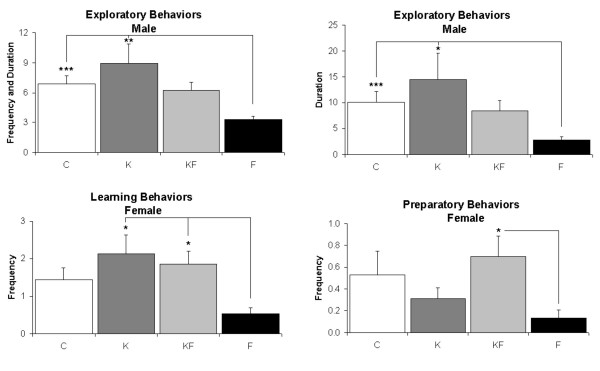
**Behavioral clusters in adult males and females in the Radial Arm Maze using DNMS paradigm**. Control (C), Ketamine (K), Ketamine and Formalin (KF), Formalin (F). Frequency denotes the number of times a behavior was noted, Duration denotes the time (in seconds) that these behaviors were maintained; upper left panel data were derived from both frequency and duration of exploratory behaviors. Bars indicate Mean ± SEM; *** P < 0.001; ** P < 0.01; * P < 0.05.

Using a delayed non-match to sample (DNMS) paradigm in the radial arm maze, the frequency and duration of various behaviors employed during the time required for consuming the bait were recorded. For the results of factorial ANOVA on individual behaviors at the 1 hour and 3 hour intervals, please see Additional files 1 and 2, respectively. Cluster analysis of the frequencies and durations of various behaviors were clustered into five groups arbitrarily described as exploratory, learning, preparatory, consumptive, and foraging (see table 1). Altered behavior patterns occurred in young adulthood following repetitive neonatal inflammatory pain in F, with reduced exploratory behaviors in the adult males (vs. C, K), as well as decreased learning (vs. K, KF) and preparatory behaviors (vs. KF) in the adult females (fig. 6). For group differences between all the patterns of behaviors identified by Cluster Analysis, please see Additional file 3.

**Table 1:** Description of Behaviors

**Frequency ** **(number of observed events)**	**Duration ** **(seconds spent in observed behaviors)**
***Exploratory Behaviors***	

Total rearing events	Total time in rearing posture
Rearing events occurring in baited arms	Total time in rearing posture in baited arms
Bait encountering events without consumption	Time in proximity of the bait without consumption
Entries into the baited arms	
Entries into all arms	

***Preparatory Behaviors***	

Rearing events occurring in the center of the maze	Time in rearing posture at the center of the maze
Total grooming events	Time to consume 75% of the bait
	Time to consume 50% of the bait
	Time to consume 25% of the bait

***Learning Behaviors***	

Re-entries into bait eaten arm after consumption	Time in bait eaten arms after full consumption
	Time in baited arms

***Consumptive Behaviors***	

Total eating events	Time spent in eating events

***Foraging Behaviors***	

Total entries in non-baited arms	Time in non-baited arms
Rearing events in non-baited arms	Total time in all arms
	Time in rearing posture while in non-baited arms
	Time to consume 100% of the bait

## Discussion

Neonatal exposure to repetitive inflammatory pain contributes to greater cell death, reduced Fos expression, and glial activation. These changes are subsequently associated with altered patterns of behavior in adult male and female rats. Neuroanatomical differences are commonly associated with cognitive outcomes following premature birth [19-21], although it remains unclear how these early developmental events alter the immature brain, leading to the cognitive differences between children born preterm or at term [22,23]. One aspect, amongst many others, may be the exposure to repetitive pain and inflammation during neonatal intensive care [23].

Rat pups experienced repeated inflammatory pain resulting from formalin injections and showed substantial elevations in cell death from many cortical regions, with several-fold differences from controls in the somatosensory cortex (Par1: 5.4-fold; Par2:12.2-fold), in the hindlimb area (8.7-fold), the frontal (5.7-fold), retrosplenial (3.3-fold), piriform (3.1-fold), occipital (1.7-fold), and temporal (1.6-fold) cortical areas. Neonatal cellular staining at 18–20 hours after the last stimulus further showed a loss of Fos expression, suggesting reduced neuronal activation from environmental inputs or reduced inputs from the cortical and subcortical areas showing increased cell death [24]. Typically, for both cellular and behavioral data sets, the F group showed greater statistical differences from controls than the K and KF groups.

Some of these changes are likely related to the glial activation following inflammatory pain (fig. 3). Both K and F treatments appeared to increase GFAP expression, although their effects were not additive. Glial localization of GFAP expression was confirmed by immunohistochemical staining, but in limited areas only. Similar to its effects in prenatal stress or systemic inflammation [25,26], glial activation may trigger a cytokine response, which contributes to the cell death occurring in widespread areas of the brain. Ketamine not only blocks NMDA receptors, but also has anti-inflammatory effects on glial, neuronal, and endothelial cells.

The minimal differences occurring between the randomized groups in the expression of caspase-3 or other apoptotic proteins suggested that cell death may be mediated via non-apoptotic pathways. This hypothesis, though unproven by the current data, is further suggested by the reduced cell death following NMDA receptor blockade (KF vs. F, fig. 1, 2), and inverse correlations between apoptotic protein expression and cell death in the F group (fig. 5). Relative expressions of Bax (considered neurodegenerative) to Bcl-2 (considered neuroprotective) correlated with neuroactivation in F; positive correlations occurred between Bax and Fos occurred in the somatosensory (Par 1), hindlimb and occipital cortical areas, whereas cleavage products of Caspase-3 correlated with Fos in the Piriform cortex. These data suggest that 3-fold increases in cortical cell death following inflammatory pain may be due to NMDA-receptor mediated excitotoxicity, occurring at a stage of brain development preceding the switch of NMDA-subunit cortical expression from NR2B to NR2A [27].

Characteristic cortical patterns of cellular activation and death for each of the four treatment groups (C, K, KF, F) are likely to change cortical development and subsequent behavior. Based on the variability of all measured behaviors (frequency and duration for each), cluster analyses grouped these into five functionally relevant domains, i.e., exploratory, foraging, consumptive, preparatory and learning behaviors. Whereas the cortex is not required for associative learning in neonatal rat pups [28], adult rats require higher cortical functions for cognition, learning and memory. The undisturbed controls utilized both visual-spatial and locomotor behaviors associated with routine exploration and position recall. Cortical cell death in the frontal and parietal association areas in F males manifested as diminished exploratory behaviors as noted previously [9] and required increased time for bait consumption [24]. Adult males in the F group spent less time in rearing behavior for visual-spatial cues and made fewer entries into the arms of the radial maze. Following preterm birth, preschool boys also show diminished exploratory behaviors in novel environments [29]. Females also had reduced total locomotor activity, reduced central rearing and grooming behaviors perhaps secondary to the neonatal cell death noted in the RSG. Cortical projections of the frontal eye fields connect the frontal, retrosplenial (RSG), and limited parietal areas to form circuits that are involved in orienting, exploring behaviors, and eye movement [30]. The RSG and RSA play a role in spatial navigation [31]. Temporary inactivation of the retrosplenial cortex impairs radial arm maze performance in the dark, impairs spatial learning during initial light training, and this inactivation alters hippocampal "place fields" as detected by recordings of complex spike cells from the hippocampus [32]. Gender differences in these long-term behavioral outcomes may result from different mechanisms of cell death in early life [33], or differences in pain-induced responses [34], or the timing of hippocampal and hypothalamic maturation due to early neurohormonal differences [35].

## Conclusion

Ketamine appears to attenuate the impaired cognitive behaviors resulting from accentuated cell death in the cortical and hippocampal fields of the neonatal rats exposed to repetitive inflammatory pain. The cellular death noted in eleven different cortical regions does not appear to be principally dependent on the apoptosis associated proteins Caspase-3, Bax, Bcl-2, or PARP, but was correlated with glial activation, as indicated by GFAP expression. The analgesic and anti-inflammatory effects of Ketamine may be neuroprotective in the setting of neonatal inflammatory pain, associated with long-term effects on adult cognitive behaviors.

## Competing interests

The authors declare that they have no competing interests.

## Authors' contributions

All authors 1) have made substantial contributions to conception and design, acquisition of data, analysis and interpretation of data; 2) have been involved in drafting the manuscript or revising it critically for important intellectual content; and 3) have given final approval of the manuscript's version being submitted.

## Supplementary Material

Additional file 1 Group Differences between Individual Behaviors at the 1 hour Interval (to test short-term learning). The data provided represent the statistical analysis of individual behaviors from factorial ANOVA values between the four randomized groups. Click here for file

Additional file 2 Group Differences between Individual Behaviors at the 3 hour Interval (to test longer-term learning). The data provided represent the statistical analysis of individual behaviors from factorial ANOVA values between the four randomized groups. Click here for file

Additional file 3 Group differences between the patterns of behaviors identified by Cluster Analysis. The data provided represent the cluster analysis of all patterns of behaviors, combining the 1 and 3 hour intervals, showing differences between the four randomized groups. Click here for file

## References

[B1] Whitfield MF, Grunau RE (2000). Behavior, pain perception, and the extremely low-birth weight survivor. Clin Perinatol.

[B2] Botting N, Powls A, Cooke RW, Marlow N (1997). Attention deficit hyperactivity disorders and other psychiatric outcomes in very low birthweight children at 12 years. Journal of Child Psychology & Psychiatry & Allied Disciplines.

[B3] Coplan JD, Andrews MW, Rosenblum LA, Owens MJ, Friedman S, Gorman JM, Nemeroff CB (1996). Persistent elevations of cerebrospinal fluid concentrations of corticotropin-releasing factor in adult nonhuman primates exposed to early-life stressors: implications for the pathophysiology of mood and anxiety disorders. Proceedings of the National Academy of Sciences of the United States of America.

[B4] Jacobson B, Bygdeman M (1998). Obstetric care and proneness of offspring to suicides as adults: case-control study. British Medical Journal.

[B5] Salk L, Lipsitt LP, Sturner WQ, Reilly BM, Levat RH (1985). Relationship of maternal and perinatal conditions to eventual adolescent suicide. Lancet.

[B6] Simons SH, van Dijk M, Anand KJS, Roofthooft D, van Lingen RA, Tibboel D (2003). Do we still hurt newborn babies? A prospective study of procedural pain and analgesia in neonates. Arch Pediatr Adolesc Med.

[B7] Chahal H, D'Souza SW, Barson AJ, Slater P (1998). Modulation by magnesium of N-methyl-D-aspartate receptors in developing human brain. Arch Dis Child Fetal Neonatal Ed.

[B8] Rabinowicz T, de Courten-Myers GM, Petetot JM, Xi G, de los Reyes E (1996). Human cortex development: estimates of neuronal numbers indicate major loss late during gestation. J Neuropathol Exp Neurol.

[B9] Anand KJS, Coskun V, Thrivikraman KV, Nemeroff CB, Plotsky PM (1999). Long-term behavioral effects of repetitive pain in neonatal rat pups. Physiol Behav.

[B10] Sternberg WF, Ridgway CG (2003). Effects of gestational stress and neonatal handling on pain, analgesia, and stress behavior of adult mice. Physiol Behav.

[B11] Anand KJS, Runeson B, Jacobson B (2004). Gastric suction at birth associated with long-term risk for functional intestinal disorders in later life. J Pediatr.

[B12] Peters JW, Schouw R, Anand KJS, van Dijk M, Duivenvoorden HJ, Tibboel D (2005). Does neonatal surgery lead to increased pain sensitivity in later childhood?. Pain.

[B13] Grunau RE, Weinberg J, Whitfield MF (2004). Neonatal procedural pain and preterm infant cortisol response to novelty at 8 months. Pediatrics.

[B14] Jevtovic-Todorovic V, Hartman RE, Izumi Y, Benshoff ND, Dikranian K, Zorumski CF, Olney JW, Wozniak DF (2003). Early exposure to common anesthetic agents causes widespread neurodegeneration in the developing rat brain and persistent learning deficits. J Neurosci.

[B15] Ikonomidou C, Bosch F, Miksa M, Bittigau P, Vockler J, Dikranian K, Tenkova TI, Stefovska V, Turski L, Olney JW (1999). Blockade of NMDA receptors and apoptotic neurodegeneration in the developing brain. Science.

[B16] Schmued LC, Hopkins KJ (2000). Fluoro-Jade B: a high affinity fluorescent marker for the localization of neuronal degeneration. Brain Research.

[B17] Walsh TJ, Chrobak JJ (1987). The use of the radial arm maze in neurotoxicology. Physiol Behav.

[B18] Boast CA, Walsh TJ, Bartolomeo AC, JJ B (2001). The delayed non-match-to-sample radial arm maze task: Application to models of Alzheimers disease. Methods of Behavior Analysis in Neuroscience.

[B19] Luciana M, Lindeke L, Georgieff M, Mills M, Nelson CA (1999). Neurobehavioral evidence for working-memory deficits in school-aged children with histories of prematurity. Dev Med Child Neurol.

[B20] Peterson B, Vohr B, Staib L, Cannistraci C, Dolberg A, Schneider K, Katz K, Westerveld M, Sparrow S, Anderson A, Duncan CC, Makuch RW, Gore JC, Ment LR (2000). Regional brain volume abnormalities and long-term cognitive outcome in preterm infants. JAMA.

[B21] Peterson BS, Vohr B, Kane MJ, Whalen DH, Schneider KC, Katz KH, Zhang H, Duncan CC, Makuch R, Gore JC, Ment LR (2002). A functional magnetic resonance imaging study of language processing and its cognitive correlates in prematurely born children. Pediatrics.

[B22] Bhutta AT, Cleves MA, Casey PH, Cradock MM, Anand KJS (2002). Cognitive and behavioral outcomes of school-aged children who were born preterm: a meta-analysis. JAMA.

[B23] Bhutta AT, Anand KJS (2001). Abnormal cognition and behavior in preterm neonates linked to smaller brain volumes. TINS, discussion 131–122.

[B24] Anand KJS, Garg S, Rovnaghi CR, Narsinghani U, Bhutta AT, Hall RW (2007). Ketamine reduces the cell death following inflammatory pain in newborn rat brain. Pediatric Research.

[B25] Semmler A, Okulla T, Sastre M, Dumitrescu-Ozimek L, Heneka MT (2005). Systemic inflammation induces apoptosis with variable vulnerability of different brain regions. J Chem Neuroanat.

[B26] Barros VG, Duhalde-Vega M, Caltana L, Brusco A, Antonelli MC (2006). Astrocyte-neuron vulnerability to prenatal stress in the adult rat brain. J Neurosci Res.

[B27] Haberny KA, Paule MG, Scallet AC, Sistare FD, Lester DS, Hanig JP, Slikker W (2002). Ontogeny of the N-methyl-D-aspartate (NMDA) receptor system and susceptibility to neurotoxicity. Toxicol Sci.

[B28] Landers MS, Sullivan RM (1999). Vibrissae-evoked behavior and conditioning before functional ontogeny of the somatosensory vibrissae cortex. J Neurosci.

[B29] Breslau N, Chilcoat H, DelDotto J, Andreski P, Brown G (1996). Low birth weight and neurocognitive status at six years of age. Biological Psychiatry.

[B30] Guandalini P (1998). The corticocortical projections of the physiologically defined eye field in the rat medial frontal cortex. Brain Res Bull.

[B31] Harker KT, Whishaw IQ (2002). Impaired spatial performance in rats with retrosplenial lesions: importance of the spatial problem and the rat strain in identifying lesion effects in a swimming pool. J Neurosci.

[B32] Cooper BG, Mizumori SJ (2001). Temporary inactivation of the retrosplenial cortex causes a transient reorganization of spatial coding in the hippocampus. Journal of Neuroscience.

[B33] Du L, Bayir H, Lai Y, Zhang X, Kochanek PM, Watkins SC, Graham SH, Clark RS (2004). Innate gender-based proclivity in response to cytotoxicity and programmed cell death pathway. J Biol Chem.

[B34] Butkevich IP, Barr GA, Vershinina EA (2007). Sex differences in formalin-induced pain in prenatally stressed infant rats. Eur J Pain.

[B35] Papaioannou A, Dafni U, Alikaridis F, Bolaris S, Stylianopoulou F (2002). Effects of neonatal handling on basal and stress-induced monoamine levels in the male and female rat brain. Neuroscience.

