# Supplementary material for: Ketamine analgesia for inflammatory pain in neonatal rats: a factorial randomized trial examining long-term effects
Source: Behav Brain Funct. 2008 Aug 7;4:35. doi: 10.1186/1744-9081-4-35 (PMC2527299; doi:10.1186/1744-9081-4-35)
Supplement: Additional file 3 — Group differences between the patterns of behaviors identified by Cluster Analysis. The data provided represent the cluster analysis of all patterns of behaviors, combining the 1 and 3 hour intervals, showing differences between the four randomized groups. [file 1744-9081-4-35-S3.doc]

**Additional File 3**

**Group Differences between Patterns of Behaviors identified by Cluster Analysis**

| **Behavior patterns** | **Male** | | | **Female** | | |
| --- | --- | --- | --- | --- | --- | --- |
|  | **Collapsed** | **Frequency** | **Duration** | **Collapsed** | **Frequency** | **Duration** |
| **Exploratory** | **P < 0.0001** | **P = 0.0735** | **P = 0.0002** | **P = 0.3141** | **P = 0.3290** | **P = 0.7070** |
|  | C vs. F, P<0.001 |  | C vs. F, P<0.001 |  |  |  |
|  | K vs. F, P<0.01 |  | K vs. F, P<0.05 |  |  |  |
| **Learning** | **P = 0.6062** | **P = 0.4598** | **P = 0.5946** | **P = 0.0681** | **P = 0.0089** | **P = 0.2997** |
|  |  |  |  |  | K vs. F, P<0.05 |  |
|  |  |  |  |  | KF vs. F, P<0.05 |  |
| **Preparatory** | **P = 0.7058** | **P = 0.1671** | **P = 0.0828** | **P = 0.6597** | **P = 0.0413** | **P = 0.5547** |
|  |  |  |  |  | KF vs. F, P<0.05 |  |
|  |  |  |  |  |  |  |
| **Consumptive** | **P = 0.3353** | **P = 0.1995** | **P < 0.0001** | **P = 0.6576** | **P = 0.2096** | **P = 0.1434** |
|  |  |  | C vs. KF, P<0.001 |  |  |  |
|  |  |  | K vs. KF, P<0.01 |  |  |  |
| **Foraging** | **P = 0.5944** | **P = 0.5177** | **P = 0.6104** | **P = 0.2102** | **P = 0.2022** | **P = 0.2432** |

P-values in **bold font** are related to ANOVA between the four randomized groups. *Post hoc* analyses to locate differences between groups were only performed if the ANOVA showed P<0.05. Randomized groups are denoted by letter symbols: C= control, K= ketamine, F= formalin, KF= ketamine-formalin.
